# 血管内大B细胞淋巴瘤诊治中国专家共识（2023年版）

**DOI:** 10.3760/cma.j.issn.0253-2727.2023.03.001

**Published:** 2023-03

**Authors:** 中华医学会血液学分会淋巴细胞疾病学组 中国临床肿瘤学会（CSCO）淋巴瘤专家委员会

血管内大B细胞淋巴瘤（intravascular large B cell lymphoma，IVLBCL）是非霍奇金淋巴瘤的一种罕见亚型，发病率约0.5/100万。与其他结外淋巴瘤不同，IVLBCL的特点为大量肿瘤细胞聚集在小或中等大小血管管腔，尤其以毛细血管和毛细血管后微静脉多见，易侵犯神经系统、皮肤、骨髓和肺等器官，而淋巴结较少受累。2008年WHO淋巴造血组织肿瘤分类将其明确归类为结外弥漫大B细胞淋巴瘤（DLBCL）的一个独立亚型[1]，2022年WHO第五版淋巴造血组织肿瘤新分类（以下简称新分类）仍然保留该亚型[2]。既往资料显示，IVLBCL患者中位生存期约1年，预后极差[3]。由于IVLBCL临床少见，对该疾病的认识尚未统一，诊治尚不规范。为加强我国临床医师对IVLBCL的认知，提高诊断、鉴别诊断及治疗水平，中华医学会血液学分会淋巴细胞疾病学组、中国临床肿瘤学会（CSCO）淋巴瘤专家委员会组织相关专家参考2007年国际结外淋巴瘤研究组制定的IVLBCL临床实践指南[4]并结合国内外最新的研究进展，讨论并制定本共识。

一、概述

IVLBCL由Pfleger和Tappeiner于1959年首次描述，当时被称为血管内皮系统性增生性疾病。1986年，Sheibani等通过免疫组织化学法证实血管内异型细胞为淋巴细胞起源，将其描述为血管内大细胞淋巴瘤。IVLBCL的中位发病年龄为64岁，男性稍多见，全身各个脏器均可侵犯[3]。病毒感染（如EB病毒）在IVLBCL发生中的作用仍需进一步确认[5]–[6]。IVLBCL细胞跨血管迁移和归巢机制缺陷导致其生长主要局限于血管内壁层，呈现出与其他类型淋巴瘤不同的临床表现和不良结局[7]–[9]。

二、临床表现

IVLBCL患者半数以上有B症状（不明原因发热、盗汗和消瘦等）。因肿瘤细胞易侵犯全身各个器官的小或中等大小血管，患者可出现因血管阻塞所致一系列复杂多变的症状，是临床误诊、漏诊的重要原因。一项回顾性研究显示，我国185例IVLBCL中约62％的患者累及2个及以上器官，首发症状高度多样化[10]。新分类根据临床表现将IVLBCL分为三个亚型：经典亚型（classic subtype）、皮肤亚型（cutaneous subtype）和噬血细胞相关亚型（haemophagocytic subtype）。

经典亚型常见于西方国家，症状与主要受累器官有关。50％～70％的患者出现不明原因的发热、疼痛、局部器官特异性症状或多器官衰竭。30％～40％的患者有中枢神经系统症状，主要为脑循环障碍所致局灶性神经功能缺损，因受累部位不同而表现各异，类似于多灶性脑血管病变、脑梗死、脊髓和神经根病变、亚急性脑病及外周神经疾病或脑神经疾病等[11]。皮肤是经典亚型最常见的受累部位（30％～40％），但多同时伴有其他器官受累表现，包括肾和肾上腺功能不全、肺动脉高压、低氧血症、肺栓塞或呼吸功能衰竭等[11]。即使临床上无明显的皮肤病灶，随机皮肤活检、骨髓评估以及经支气管肺活检也有助于诊断[12]–[13]。尽管肿瘤细胞位于血管腔内，但5％～10％的病例在外周血涂片中仍能发现肿瘤细胞。

皮肤亚型是指病变仅累及皮肤者。皮肤损害最常见的部位为下腹部及下肢等区域。皮损形态多样，包括“橘皮样”改变、紫色丘疹或结节、孤立或聚集呈簇的斑块和溃疡，或类似血管内栓塞病伴网状青斑样损害。该亚型患者往往表现出更有利的临床特征，包括美国东部肿瘤协作组体能状态（ECOG-PS）评分≤1分和血细胞计数正常。尽管多达30％的患者可能出现全身症状，但临床经过相对情性，不同于经典亚型[12]。

噬血细胞相关亚型多见于亚洲国家，患者常表现为多器官功能衰竭、肝脾肿大和全血细胞减少，骨髓和外周血检查可检测到肿瘤细胞。起病和临床进展均非常迅速。

三、检查

IVLBCL的实验室检查无特异性，以贫血和（或）血小板减少最常见，通常伴有乳酸脱氢酶升高和（或）低白蛋白血症。存在不能解释的血象异常时，需尽快完善多部位骨髓活检；对于不明原因发热者，排除感染、实体肿瘤及自身免疫性疾病后，需尽早对临床或影像学检查显示的可疑部位行多次多点活检。随机多部位皮肤活检（random skin biopsy, RSB）（强调必需深达肌层；建议取材部位为大腿两侧和腹部）对临床疑似患者早期明确诊断意义重大[12]–[15]。一项回顾性研究显示，RSB对于诊断IVLBCL的敏感度和特异度分别为78％和99％[16]。下述临床和实验室指标对于临床尽早行RSB有指导作用，包括：不明原因发热；意识改变；低氧血症；PLT<120×10^9^/L；乳酸脱氢酶>800 U/L；血清可溶性白细胞介素2受体（sIL2R）水平>5 000 U/ml。具备5项者，预测RSB阳性概率为65％，不足2项者，RSB阳性概率为0[16]。

近年来，细胞因子检测在协助特殊部位淋巴瘤的诊治中发挥了重要作用[17]。北京协和医院的研究表明，血清白细胞介素10>95.65 pg/ml时，诊断IVLBCL的敏感度和特异度分别为80％及100％，且可用于疗效监测[18]。临床出现诊断困难时，应进行细胞因子检测，用于协助诊断与鉴别诊断。

病变早期影像学检查无特异性表现，中晚期可在影像学上表现为肿瘤占位效应，提示IVLBCL存在血管外浸润可能，并非绝对血管内生长[19]。根据具体临床表现可以选择颅脑增强MRI、胸腹部增强CT和正电子发射断层扫描（PET-CT）等检查。中枢神经系统受累患者的颅脑影像提示肿瘤多累及额、顶、颞、枕叶，可表现为占位效应、低密度灶或腔隙性脑梗死和出血，也可仅表现为信号异常[20]。肺部受累患者，肺部CT可表现为双肺多发磨玻璃影、结节影和胸腔积液等[10]。PET-CT检查在辅助病理取材中具有重要作用，但值得注意的是，PET-CT阴性时也不能排除IVLBCL[21]–[22]。

病理检查镜下表现为单个或成簇淋巴瘤细胞分布于小或中等大小血管腔内，瘤细胞游离漂浮、黏附呈巢状或边集/黏附于血管壁生长，但血管结构尚完整；部分病例可见血管腔内纤维素性血栓、出血和坏死等现象。肿瘤细胞以大细胞为主，核圆形、类圆形或不规则，核膜常清晰，染色质较粗，核仁明显，核分裂象常易见，形态类似DLBCL细胞。少数瘤细胞呈间变特征或较小细胞形态。部分病例在血管腔外可见少量/小灶肿瘤细胞。肿瘤细胞免疫表型表达广谱B细胞标志，包括CD20、PAX5、CD19、CD22、CD79a、OTC2、BOB1等，约38％的病例表达CD5；大部分（75％～80％）病例肿瘤细胞表达MUM1/IRF4，呈非生发中心免疫表型，但有约13％的病例表达CD10。Ki-67增殖指数一般较高，约81％的患者Ki-67≥60％。部分病例肿瘤细胞表达PD-L1[23]，有研究结果提示PD-L1可能与IVLBCL的免疫逃逸有关[24]。此外，CD34和CD31标记血管内皮细胞可用于证实肿瘤细胞位于血管内。

分子遗传学检查提示IVLBCL无特异性染色体易位，免疫球蛋白（Ig）重链和轻链基因呈克隆性重排。二代测序分析提示IVLBCL为非生发中心B细胞基因表达谱系，存在NF-κB通路过度激活[25]，MYD88（57％）、CD79B（67％）、SETD1B（57％）和HLA-B（57％）突变频率较高[26]。

四、诊断、鉴别诊断、分期和预后

1. 诊断：IVLBCL的诊断主要依靠组织病理学，即小到中等血管管腔内发现大B淋巴瘤细胞。取材部位通常推荐骨髓或皮肤[27]–[28]。由于受累血管大多位于皮下脂肪层，因此必须进行深部皮肤活检。对于疑似累及脑部的患者，应在脑组织活检前进行皮肤活检[29]。如果皮肤活检不能明确诊断，特别是有证据表明存在器官功能障碍时，应活检其他部位。影像学引导下病变部位活检可提高诊断的敏感性。

2. 鉴别诊断：

（1）血管内NK/T细胞淋巴瘤：新分类已将该肿瘤作为一个独立的亚型在侵袭性NK细胞白血病章节加以描述。瘤细胞常为小至中等大小或小、中、大细胞混合存在，免疫组织化学显示肿瘤细胞表达胞质CD3、CD56及细胞毒分子（TIA1、GranzymeB和Perforin），CD5常阴性，通常不表达B细胞标志物，与EB病毒感染密切相关[30]。

（2）炎性病变：IVLBCL可表现为类似急性肝胆感染的临床表现[31]或粟粒性肺结核的相关特征[32]，需引起临床重视，以免漏诊或误诊。

（3）DLBCL累及血管及淋巴管：DLBCL有时会在血管及淋巴管腔内形成瘤栓，但同时伴有明显的血管外肿块，如淋巴结受累肿大或结外脏器占位性病变，需结合临床病史、影像学检查及组织病理诊断鉴别。

（4）转移性癌：成簇的癌细胞位于淋巴管和（或）血管内；免疫组织化学染色CK阳性而白细胞共同抗原阴性，易于鉴别。

3. 分期：目前尚无IVLBCL特有的分期体系，仍采用Ann Arbor分期系统。大多数病例诊断时即为Ⅳ期[3]。既往报道仅有皮肤累及，分期为Ⅰ_E_期的病例，尸检均证实为Ⅳ期。因此，对于诊断时存在不能解释的脏器功能不全，需要警惕器官累及可能。

五、治疗

目前尚无最佳的治疗方案，通常采用以R-CHOP方案为基础的联合化疗，1年、3年的总生存（OS）率分别为60％、17％，中位OS时间为450 d，中位无进展生存（PFS）时间为420 d。

中枢神经系统累及是预后不良的重要因素[3]，初诊时伴有中枢神经系统受累患者，1年内中枢神经系统复发概率为25％；而初诊时不伴中枢神经系统受累患者，3年内中枢神经系统复发概率为25％[33]，因此预防中枢神经系统复发至关重要[34]。PRIMEUR-IVL研究是一个多中心的、单臂的、Ⅱ期临床试验，评估R-CHOP方案联合大剂量甲氨蝶呤和鞘内注射对初诊且无中枢神经系统受累IVLBCL患者的疗效和安全性。中位随访3.9（2.5～5.5）年，2年PFS率和OS率分别为76％和92％，2年内中枢神经系统复发风险为3％[35]，国内北京协和医院的研究同样证实了上述疗效[18]。基于此，本共识推荐R-CHOP方案联合大剂量甲氨蝶呤的治疗作为初诊且无中枢神经系统受累IVLBCL患者的一线诱导方案。当前的治疗策略，包括鞘内注射、大剂量甲氨蝶呤等药物的应用对诊断时伴有中枢神经系统受累患者的预后改善仍然有限，亟需探索新的治疗策略[3]。

基于IVLBCL患者MYD88、CD79B突变频率高的特征[26]，R-CHOP方案联合BTK抑制剂成为目前探索的方向。北京协和医院一项前瞻性、单臂、Ⅱ期临床试验评估泽布替尼联合R-CHOP（ZR-CHOP）方案治疗初诊IVLBCL患者的疗效和安全性，截至2021年7月共入组9例患者，均获得治疗反应，其中5例取得完全缓解，中位随访10（1～18）个月时，疗效持续保持，且安全性可控，值得临床推广应用[36]。

IVLBCL患者复发后的生存极差，如何避免复发是临床关注的另一问题。缓解后序贯自体造血干细胞移植（ASCT）巩固治疗的IVLBCL患者的3年PFS率和OS率分别为83％和89％，3年累积复发率为14％[37]–[38]。因此，本共识推荐对于年龄小于65岁或体能状况较好的患者行ASCT巩固治疗。

六、疗效评估和随访

与其他类型淋巴瘤不同，IVLBCL的疗效评估标准为[27]：①完全缓解：诊断时所有临床症状、实验室检查异常指标均正常，并且无任何新发表现；②疾病进展：出现与疾病有关的新发异常，或与疾病相关的初始异常明显恶化；③疾病稳定：未获得完全缓解，也无疾病进展；④疾病复发：最初获得完全缓解后病情进展。

随访频率通常参考其他类型淋巴瘤，即完成治疗后的前2年应每3个月进行一次随访，包括病史、查体、血细胞计数、生化常规和细胞因子检查，每6个月进行一次影像学检查（包括头颅、颈部、胸部及全腹部）。完成治疗后第3～5年每半年进行一次随访。5年后每年进行一次随访或有症状时进行检查，如有异常或考虑疾病进展，则进行相关的检查等。

七、预后

IVLBCL的预后主要与其累及部位相关，单纯皮肤累及患者的预后常相对较好；诊断时表现为中枢神经系统受累及合并噬血细胞综合征的IVLBCL患者预后差。IVLBCL的预后与患者治疗与否也明显相关。年龄>60岁、未接受大剂量甲氨蝶呤治疗及未获得完全缓解均是不良预后因素[18]，目前研究数据并未发现分子特征和疾病预后的相关性[1],[3]。
